# Protein Profiling in Hepatocellular Carcinoma by Label-Free Quantitative Proteomics in Two West African Populations

**DOI:** 10.1371/journal.pone.0068381

**Published:** 2013-07-30

**Authors:** Haddy K. S. Fye, Cynthia Wright-Drakesmith, Holger B. Kramer, Suzi Camey, Andre Nogueira da Costa, Adam Jeng, Alasana Bah, Gregory D. Kirk, Mohamed I. F. Sharif, Nimzing G. Ladep, Edith Okeke, Pierre Hainaut, Simon D. Taylor-Robinson, Benedikt M. Kessler, Maimuna E. Mendy

**Affiliations:** 1 Nuffield Department of Medicine, Henry Wellcome Building for Molecular Physiology - University of Oxford, Oxford, Oxfordshire, United Kingdom; 2 Laboratory Services and Bio-bank Group, International Agency for Research on Cancer, Lyon, France; 3 Department of Disease Control and Elimination, MRC Unit (UK) The Gambia Laboratories, Fajara, Banjul, The Gambia; 4 Department of Epidemiology - Bloomberg School of Public Health, Johns Hopkins University, Baltimore, Maryland, United States of America; 5 Departamento de Estatistica, Instituto de Matematica, Universidade Federal do Rio Grande do Sul, Rio Grande, Brazil; 6 Liver Unit - Division of Diabetes Endocrinology and Metabolism, Department of Medicine, Imperial College London, London, United Kingdom; 7 The International Prevention Research Institute, Lyon, France; 8 Jos University Teaching Hospital, Jos, Plateau State, Nigeria; Drexel University College of Medicine, United States of America

## Abstract

**Background:**

Hepatocellular Carcinoma is the third most common cause of cancer related death worldwide, often diagnosed by measuring serum AFP; a poor performance stand-alone biomarker. With the aim of improving on this, our study focuses on plasma proteins identified by Mass Spectrometry in order to investigate and validate differences seen in the respective proteomes of controls and subjects with LC and HCC.

**Methods:**

Mass Spectrometry analysis using liquid chromatography electro spray ionization quadrupole time-of-flight was conducted on 339 subjects using a pooled expression profiling approach. ELISA assays were performed on four significantly differentially expressed proteins to validate their expression profiles in subjects from the Gambia and a pilot group from Nigeria. Results from this were collated for statistical multiplexing using logistic regression analysis.

**Results:**

Twenty-six proteins were identified as differentially expressed between the three subject groups. Direct measurements of four; hemopexin, alpha-1-antitrypsin, apolipoprotein A1 and complement component 3 confirmed their change in abundance in LC and HCC versus control patients. These trends were independently replicated in the pilot validation subjects from Nigeria. The statistical multiplexing of these proteins demonstrated performance comparable to or greater than ALT in identifying liver cirrhosis or carcinogenesis. This exercise also proposed preliminary cut offs with achievable sensitivity, specificity and AUC statistics greater than reported AFP averages.

**Conclusions:**

The validated changes of expression in these proteins have the potential for development into high-performance tests usable in the diagnosis and or monitoring of HCC and LC patients. The identification of sustained expression trends strengthens the suggestion of these four proteins as worthy candidates for further investigation in the context of liver disease. The statistical combinations also provide a novel inroad of analyses able to propose definitive cut-offs and combinations for evaluation of performance.

## Introduction

Hepatocellular carcinoma (HCC) accounts for 85–90% of all tumours emerging from the liver in high incidence areas such as Asia, Sub-Saharan Africa and parts of Eastern Europe, and between 70–75% of cases in lower incidence regions. It is the fifth most common cause of cancer related mortality worldwide for males and seventh for females [1]. The main reason for the disproportionate spread of HCC is attributable to the prevalence of its major risk factors, that is, chronic infection with the Hepatitis B Virus (HBV), Hepatitis C Virus (HCV) [2] and exposure to Aflatoxin B1 (AFB1) [3] which exist in the developing world. It has a poor prognosis, making it the overall third highest cause of cancer related mortality worldwide [4]. In a global survey overseen by the World Health Organisation (WHO), it was reported that there were approximately 598,000 deaths per annum (pa) attributable to Liver Cancer [5]. One of the key reasons for this is the lack of a low cost, reliable early diagnostic and screening test, useable in the developing world where access to high performance compensatory diagnostic aids are severely limited. The lack of viable and affordable treatment options in the developing world is also a significant contributing factor to the poor prognosis of this condition.

Primary HCC is a complex multistep disease, which arises from a myriad of environmental, host genetic and viral factors. Up to 10% of individuals who become infected with the HBV will be unable to clear it and become chronic carriers. From this, a fraction will develop HCC, with or without liver cirrhosis (LC) [6] with a fraction of them not displaying viral antigens in their sera (occult hepatitis) [7]. These observations support suggestions that HBV is itself a direct trigger for HCC as it has been shown to incorporate into host Deoxyribonucleic Acid (DNA) in up to 80% of cases [8]–[10]. This is in contrast to HCV induced HCC for which strong evidence suggestive of direct viral effects has been difficult to come by [11], [12]. AFB1, is another major causative element linked to HCC and is produced by the fungi *aspergillus flavus* contaminating poorly stored grains and nuts. In populations with large exposure to this toxin, research shows the risk of HCC development to be in direct proportion to the amount of aflatoxin ingested [13], [14]. This is especially highlighted in a Chinese study showing the relative risk of HCC development as 3.4 in individuals exposed to aflatoxin and 7.3 in those chronically infected with HBV. In patients with both factors present, the relative risk of HCC development rose significantly to 59.4 [3], [15]. As HCC is a multifactorial disease, the combination of chronic viral infection and ingestion of the toxin work synergistically to increase the risk of disease development.

Mass spectrometry (MS) and proteomics approaches have been used to propose a growing list of biomarker candidates for diagnostic and prognostic use in HCC as reviewed in recent publications [16], [17]. For instance, studies on HCC tissue [18], [19] and serum [20], [21] have revealed a number of molecular candidates that are currently at various stages of early clinical validation. To date, however, AFP is the main non-invasive clinical marker used in the diagnosis and therapeutic monitoring of this disease, despite its limited specificity and sensitivity [22], [23]. To counter this, a comprehensive study using plasma samples from HCC & HBV endemic areas was launched, setting out to use proteomic fingerprinting methods to identify highly sensitive and specific biomarker candidates of greater performance than AFP.

Twenty-six differentially expressed proteins were identified from discovery MS experiments with six of these further validated in individual subject plasma and four displaying results suggestive of their plausibility as novel biomarker candidates for HCC. To develop shortlisted protein biomarker candidates and confirm observed differences, independent method and subject validations were conducted and the results incorporated into extensive statistical analyses. This work represents the first extensive protein biomarker study conducted on West African cohorts.

## Methods

### Ethics Statement

All subjects recruited into the GLCS gave written informed consent prior to inclusion. Written informed consent was also provided by all JUTH subjects. Final approval and authorization for use of the Nigerian samples in research experiments was given by the research and ethics committee of the University of Jos.

The overall ‘Biomarker Discovery in West African populations’ study proposal was approved by the joint Gambia Government/MRC Unit and London School of Hygiene and Tropical Medicine Scientific Coordinating and Ethics Committees (SCC & Ethics Committee Approval # 1154).

Both subject populations were recruited in concordance with the ethical guidelines presented in the Declaration of Helsinki.

### Study Groups

Two distinct plasma sample sets were used in these investigations, one recruited from the Gambia; the primary source of samples for this study and the second from Nigeria; used as a regional pilot validation cohort. Briefly, the Gambia Liver Cancer Study (GLCS) samples were collected as part of a five-year liver cancer case-control study with subjects recruited from three major healthcare providers in the Gambia; Royal Victoria Teaching Hospital (RVTH), Bansang Hospital and the Medical Research Council (MRC) Unit, Fajara. Controls as well as HCC and LC cases were recruited from all three study sites.

The Nigerian samples were sourced from the Jos University Teaching Hospital (JUTH) by regional collaborators in a study overseen by colleagues at Imperial University, London.

### GLCS Subject Selection Method & Diagnosis

The GLCS population consists of subjects who were referred to these centres by local Physicians after displaying signs of liver disease as well as those who were actively sought by field worker surveillance of wards and clinics. Eligible individuals were approached regarding the study and those who were willing gave informed consent to be included (figure S*1*). Subjects were bled in EDTA tubes and in the urban health facilities had their blood samples sent to the on site Serology lab for immediate separation followed by storage at -20°C for working samples and −80°C for all remaining aliquots. The samples from Bansang Hospital were separated immediately following bleeding and frozen at −80°C on site pending transport on dry ice to the MRC Serology Lab in Fajara. Working aliquots were taken for lab analyses, which were used to profile recruited subjects as controls, LC or HCC cases based on clinical examination, AFP measurement, ultrasonography and liver biopsy. The ICD 10 staging standard was applied to HCC cases that had liver biopsies performed but this was not taken into consideration when pooling due to the small fraction of overall HCC cases that had confirmatory liver biopsies done. An ultrasound (US) scoring system [24], [25] was used in the diagnosis of LC & HCC. This was based on ultrasonographic examinations of the liver surface, parenchyma, vascular structure and spleen compared against the gold standard of liver biopsy. In subjects that have developed LC from chronic HBV infection; sensitivity and specificity values of 77.8% and 92.5% respectively have been attained. Approximately 24% of the subjects diagnosed as having HCC in the GLCS population underwent a liver biopsy (table 1).

**Table 1:** Summary of key clinical parameters in GLCS subject population.

Variable	Control No.	Control %	LC No.	LC %	HCC No.	HCC %
**Biopsy**	n/a	n/a	5	5.1	29	24.2
**Clinica Exam**	120	100	99	100	120	100
**Ultrasound**	n/a	n/a	99	100	120	100
**AFP>100 ng/ml**	0	0	20 ^of 95 tested^	21.1	106	88.3
**Mean ALT (IU/L)**	6.58	n/a	12.4	n/a	14.3	n/a
**Mean AST (IU/L)**	19.3	n/a	64.5	n/a	158.4	n/a
**ALT>56 IU/L**	1	94.2 tested	0	92.3 tested	4	92.5 tested
**AST>40 IU/L**	8	93.3 tested	46	94.0 tested	83	94.2 tested
**Male**	84	70	63	63.6	92	76.7
**Female**	36	30	36	36.4	28	23.3
**Age (mean)**	44.4	n/a	43.7	n/a	49.1	n/a
**HbsAg Positive**	10^of 119 tested^	8.4	54 ^of 99 tested^	54.5	65 ^of 117 tested^	55.6
**Tp53 Mut Positive**	5 ^of 113 tested^	4.4	11 ^of 89 tested^	12.4	38 ^of 105 tested^	36.2
**E Antigen Positive**	0^ of 10 positive^	0	13 ^of 41 tested^	31.7	12 ^of 53 tested^	22.6
**HCV Positive**	7 ^of 120 tested^	5.8	14^ of 97 tested^	14.4	38^ of 120 tested^	31.7

Scientific and Ethical approvals (MRC Scientific Coordinating Committee No. 1154) were granted for 339 single plasma aliquots of 0.5-1ml to be removed from the frozen archives for utilization in this study. These formed a nested selection of case-control samples from the full GLCS cohort. The nested population selection was based on statistics estimating the minimum subjects per category that would allow for significant differences in protein abundance to be identified. The GLCS subjects were not tested for any viral infections aside from HBV and HCV.

### JUTH Subject Selection & Diagnosis

The ethics committee at JUTH in Plateau state, Nigeria approved the study proposal allowing for sample collection to be completed. Eligible individuals were consented and had their blood collected into plasma EDTA tubes. In total, plasma aliquots for 55 subjects (table 2) were donated by the Taylor-Robinson group at Imperial [26] for validation experiments with these classified as either Nigerian healthy controls (NN), Nigerian Asymptomatic Carriers (NASC), Nigerian cirrhotics (NCirr) or Nigerian HCCs (NHCC). The categorizations were based on tests for HBV, HCV and Human Immunodeficiency virus (HIV) virus status, AFP, biochemical liver indices, US, or Computed Tomography (CT), endoscopy and or liver biopsy.

**Table 2:** Summary of key clinical parameters in JUTH subject population.

Variable	NN	NCLD	NCirr	NHCC
**Biopsy (%)**	0	77.7	16.7	4.8
**Clinica Exam (No.)**	10	18	6	21
**Ultrasound (%)**	100	–	83.3	100
**AFP>100 ng/ml (%)**	0	0	16.7	80.9
**Mean ALT (IU/L)**	22.8	29.6	42	73.3
**ALT>56 IU/L (No.)**	0	1	2	8
**Male (%)**	50	72.2	83.3	61.9
**Female (%)**	50	27.8	16.7	38.1
**Mean Age (years)**	41	38.1	39.2	47.7
**HbsAg Positive (%)**	0	100	100	90.5
**HCV Positive (%)**	0	0	0	4.8

Subjects who tested negative for the hepatitis B surface antigen (HBsAg), HCV antibody as well as HIV and had results not indicative of liver disease from biochemical assays for AFP, creatinine, urea, Alanine aminotransferase (ALT), alkaline phosphatase, and total bilirubin, were taken as healthy controls. Asymptomatic carrier subjects were identified as those positive only for HBsAg with no evidence of cirrhosis following liver biopsy (conducted on 80% of NASC group). Those without biopsy had US done which showed no evidence of early stage LC. The NCirr group within this population were all HBsAg positive with evidence of cirrhosis seen during US as portal hypertension. Only one subject had their LC diagnosis confirmed by liver biopsy. The HCC group had a >95% HBV positive rate with the clinical diagnosis of HCC based on AFP, US, CT and for one subject, liver biopsy.

The HCCs were assessed using the Okuda staging system [27] with all of them identified as Okuda stage II or higher.

### GLCS Sample Pooling for Label Free MS

Suspected aetiology of liver disease and age were considered as the basis for stratifying and pooling samples. Pooling subjects by age and disease categorization was opted for in the end as it provided not only more balanced sample numbers for the nine sub-groups but also allowed for the grouping of individuals with expected similar levels of basal liver functionality. Sample collection site was not taken into consideration when pooling. As a first step, 20 µl of plasma was taken from GLCS subjects in each age and disease category (figure S2) and pooled together in order to allow for the identification of robust markers not subject to individual variations. From this, 200 µl of pooled plasma was taken forth into the IgG and albumin depletions and downstream protocols as described below.

### T2 Depletion, Protein Quantitation, Off-gel Fractionation & Gel Viewing

The nine sample pools from the GLCS (figure S2) were each subjected to top 2 depletion of albumin and IgG [28]–[30]. Representative aliquots were taken from each depleted pool and subject to a protein quantitation assay using the *EZQ Protein Quantitation Kit* as per manufacturer’s instructions.

**Figure 1:**
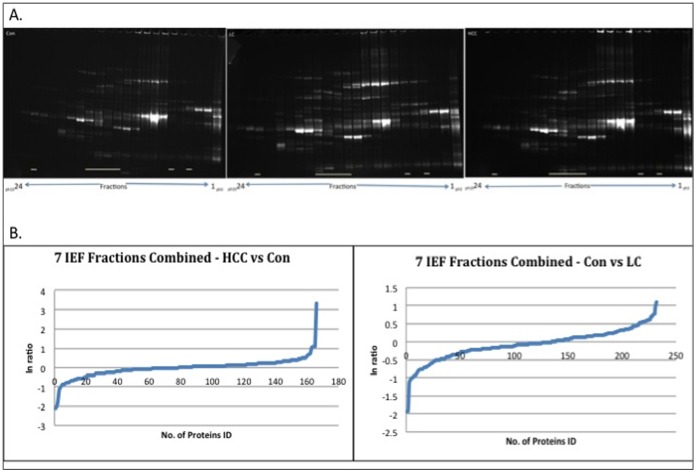
Quality control of fractionation for MS analysis. (**A**) Representative SDS-PAGE gel analysis of isoelectric focussing (IEF) fractions from each disease group following IgG & albumin depletion and off-gel fractionation of subject pools. Visualisation was performed with Sypro Ruby Red staining showing healthy control (Con, left panel), cirrhosis (middle panel) and hepatocellular carcinoma (HCC, right panel). (**B**) Natural log distribution plots of proteins showing their expression changes (ln ratio) for non-liver ailment controls versus HCC and liver cirrhosis cases.

Isoelectric focusing (*OFF-GEL Fractionator, 3100 Agilent*) into 24 fractions was performed on high-resolution pH 3–10 strips as per manufacturer’s protocol. For comparative purposes, aliquots from all 24 off-gel fractions were separated by Sodium Dodecyl Sulphate Polyacrylamide Gel Electrophoresis (SDS-PAGE) using a 4–12% Criterion precast Biorad system run at 200 V for 1 hour. The gels were fixed in 40% Methanol (MeOH) and 10% acetic acid (AA) then stained overnight with SYPRO Ruby. A destain was conducted the next day with 10% MeOH and 7% AA. The gel images were viewed on a UV transilluminator (*AutoChemi System*) with image acquisition using the *LabWorks 4.6* software. The gel images were compared for all 24 fractions and three disease groups and used to determine which fractions would be taken forward for analysis by MS. The chosen fractions are highlighted in figure 1A and were primarily picked based on the amount of protein material present, absence of an overwhelmingly abundant protein band and lane uniformity across the respective age groups.

### Protein Precipitation, Digestion, Desalting & MS Analysis

The seven selected off-gel fractions were taken forth for purification by MeOH-chloroform precipitation of proteins [31]. The obtained pellets were digested and desalted as previously described [32]. The resultant purified peptides were evaporated in a vacuum centrifuge, frozen at −80 C and resuspended in 50 µl of solution A (97.9% H2O, 2% Acetonitrile (ACN) & 0.1% Formic Acid (FA)) on the day of analysis.

The samples generated from this extensive workflow were analysed by nano-ultraperformance liquid chromatograpy electrospray ionization (nUPLC™ ESI) MS/MS in triplicate on a 75 µm inner diameter 25 cm length C18 nano-Acquity™ UPLC™ column coupled to a Quadrupole-Time Of Flight (Q-TOF) premier mass spectrometer (Waters), with 1.7 µm particle size on a 90 minute gradient of 2–45% solution B (19.9% H2O, 80% ACN and 0.1% FA). Data acquisition was performed in high-definition MS^E^ mode which utilizes high and low collision energy switching, every 1.5 seconds [33].

### Data Analysis and Quantitation

Data processing including deisotoping and deconvolution was performed using the Protein Lynx Global Server software (*PLGS v2.2.5, Waters*). MS/MS spectra were reconstructed by combining all precursor and fragment masses with identical retention times. The mass accuracy of the raw data was corrected using Glu-fibrinopeptide (200 fmol/µl; 700 nl/min flow rate; 785.8426 Da [M +2H]^2+^) that was infused into the mass spectrometer as a lock mass during analysis (every 30 seconds). Peptides and regarding proteins were identified by searching the peak lists against a UniProt/SwissProt database [version 2009.04.23; 19713 human sequence entries] using the following parameters: Minimum fragment ion matches per peptide: 3; minimum fragment ion matches per protein: 7; minimum peptide matches per protein: 1; maximum protein mass: 250000 Da; primary digest reagent: trypsin; missed cleavages: 1; fixed modifications: Carbamidomethyl (C); variable modifications: Oxidation (M). All protein hits that were identified with a confidence score of >95% were included in the quantitative analyses. Identical peptides from each triplicate set per sample were clustered based on mass precision (<10 ppm, typically ca 5 ppm) and a retention time tolerance of <0.25 min using the clustering software included in PLGS v2.3. If two or more distinct proteins shared an identical peptide but were found to be regulated differently, then the quantitation algorithm did not include the peptide in question. In order to allow for this, peptide probabilities are always softened by the PLGS software slightly prior to quantitation. Because of this, the contributions from peptides with even 100% probability of presence were suppressed in order to avoid potential errors in quantitation. Normalisation was performed using the PLGS “auto-normalization” function. The statistical significance of relative expression ratios was calculated using a Monte-Carlo algorithm and expressed as p<0.05 for down-regulated and 1−p>0.95 for up-regulated proteins, respectively. Only proteins that were identified in two out of three replicate analysis runs were selected for further analysis (replicate filter two). To simplify data analysis, multiple regulation factors obtained for one unique protein detected in distinct fractions had to be reduced to a single value. We did not consider multiplicate protein entries that had controversial regulation values throughout different fractions. For multiplicates that showed a common trend for all detected data points, the minimal regulation factor detected was chosen. A list of differentially expressed proteins was also imported into the Ingenuity Pathway Analysis (IPA) software, and the Biomarker discovery tool was consulted for the short-listing of proteins as potential marker candidates (table 3). The decision for which shortlisted markers to take forth for extensive, individual subject based validation was dependent on literature reports of the proteins in relation to HCC, commercial kit and antibody availability as well as representation of various protein families.

**Table 3:** Differentially expressed proteins in plasma from controls, LC and HCC subjects identified by quantitative MS.

Differentially Expressed Proteins	Fraction	Swissprot	ID. Score	Seq.Cov. %	♯ ofPeptides	HCC vs CON Ratio	CON vs. LC Ratio
alpha-2-macroglobulin	14	P01023	1385.68	67.5	83	1.04 (1), 1.32 (1), 1.04 (0.98)	***HCC vs. CON***
apolipoprotein E	14	P02649	391.2	71.3	27	1.42 (1), 1.60 (1), 1.40 (1)	0.92 (0.01), 0.74 (0), 0.76 (0)
complement component4 binding protein,α	14	P04003	91.8	40.4	20	1.07 (0.97), 1.68 (1), 0.96 (1)	0.97 (0.3), 0.84 (0), 0.90 (0.05)
complement factor I	14	P05156	61.02	36.7	20	1.07 (0.8), 1.07 (0.86),1.04 (0.64)	***HCC vs. CON***
glutathione peroxidase 3	14	P22352	61.26	42	10	1.27 (1), 1.45 (1), 1.04 (0.65)	0.66 (0), 0.61 (0), 0.72 (0)
apolipoprotein H	15	P02749	49.75	41.4	10	1.04 (0.61), 1.20 (0.97),1.06 (0.77)	2.39 (1), 1.77 (1), 1.80 (1)
complement component 4B	6	P0C0L5	384.56	17.1	22	1.02 (0.69), 1.68 (1), 1.04 (0.91)	1.30 (1), 1.36 (1), 1.21(1)
Alpha-1-antichymotrypsin	6	P01011	524.43	37.6	13	1.65 (1), 1.34 (1), 1.28 (1)	0.84 (0), 0.51 (0), 0.96 (0.07)
carboxypeptidase N, polypeptide 2	4	P22792	206.51	38	14	1.19 (0.93), 1.05 (0.76),1.06 (0.71)	***HCC vs. CON***
leucine-rich alpha-2-glycoprotein 1	4	P02750	170.54	49.6	15	1.86 (1), 1.40 (1), 1.11 (0.92)	0.51(0), 0.84 (0), 0.79 (0)
complement component 3	14	P01024	569.1	52.1	79	0.88 (0), 0.84 (0), 0.78 (0)	1.46 (1), 1.56 (1), 1.19 (1)
apolipoprotein A-I	15	P02647	110.79	65.9	17	0.55 (0), 0.68 (0), 0.66 (0)	1.52 (1), 1.23 (0.99), 1.73 (1)
complement factor H	15	P08603	566.61	62.5	63	0.90 (0), 0.69 (0), 0.90 (0)	1.15 (1), 1.42 (1), 1.25 (1)
haptoglobin-related protein	15	P00739	191.45	59.2	21	0.64 (0), 0.65 (0), 0.99 (0.41)	1.43 (1), 1.38 (1), 1.21(1)
hemopexin	13	P02790	333.07	59.3	23	0.56 (0), 0.45 (0), 0.68 (0)	1.57 (1), 1.92 (1), 1.60(1)
alpha-1-microglobulin/bikunin precursor	6	P02760	158.24	31	7	0.75 (0), 0.71 (0), 0.73 (0)	1.39 (1), 1.27 (1), 1.42 (1)
paraoxonase 1	6	P27169	146.28	39.4	9	0.54 (0), 0.81 (0.07), 0.54 (0)	1.27 (1), 1.52 (0.99), 1.78 (1)
clusterin	4	P10909	102.59	39.2	17	0.76 (0), 0.48 (0), 0.52 (0)	1.52 (1), 1.67 (1), 2.30 (1)
complement component 4A	15	P0C0L4	82.92	19.6	28	***CON vs. LC***	1.28 (1), 1.30 (1), 1.22 (1)
haptoglobin	13	P00738	286.29	60.1	22	***CON vs. LC***	1.54 (1), 1.35 (1), 1.17 (1)
complement factor B	21	P00751	544	52.2	36	***CON vs. LC***	2.36 (1), 1.97 (1), 1.20 (1)
Alpha-1-antitrypsin	4	P01009	186.57	39.5	14	***CON vs. LC***	0.39 (0), 0.29 (0), 0.87 (0.21)
caspase 8	13	Q14790	16.81	12.5	7	0.80 (0.1), 0.66 (0.01),0.93 (0.31)	***HCC vs. CON***
fibrinogen alpha chain	13	P02671	75.6	27.5	19	0.90 (0.04), 0.84 (0), 0.51 (0)	1.12 (0.96), 1.11 (0.93), 1.52 (1)
amyloid P component, serum	13	P02743	94.24	36.8	10	***CON vs. LC***	2.83 (1), 1.84 (1) 1.40 (1)
CD5 molecule-like	12	O43866	650.63	61.7	23	1.17 (1), 1.32 (1), 1.19 (1)	0.61 (0), 0.54 (0), 0.53 (0)

Format of tabulated results is = Ratio of compared values (p-value pertaining to it) in the order Young, Middle, Old Up regulated proteins are those with ratios>1. Cut off for significance = 0.95 (1−p) Down regulated proteins are those with ratios <1. Cut off for significance = 0.05 (1−0.95).

### ELISA Assays

Enzyme linked immunosorbent assay (ELISA) based measurements were carried out using commercial kits for the four proteins; α-1-antitrypsin (α1AT), apolipoprotein A1 (Apo A1), hemopexin (HPX), (*Immunology Consultants Laboratory, Portland, OR USA*) and complement component 3 (CC3) (*Abnova, Taiwan*) according to manufacturers instructions. All the assays were optimised and were used at the following dilution factors: α1AT at 1∶50,000; Apo A1 at 1∶20,000, HPX at 1∶30,000 and CC3 at 1∶400.

Absorbance measurements were taken at 450 nm and a 4-parameter logistic regression standard curve generated to extrapolate select protein levels in individual samples, taking into account the respective dilution factors.

### Protein Level Extrapolation & ROC Curves

To determine the diagnostic ability of these four proteins, the statistical software Graph Pad Prism (*version 5*) was used to generate Receiver Operator Characteristic (ROC) curves. The area under these ROC’s is well recognised as a measure of diagnostic ability for biomarkers [34]. All the background subtracted duplicate measurements from the known standard and unknown sample readouts were exported to Graph Pad Prism. The mean of the duplicate measurements for each subject was taken and Log_10_ transformed followed by the performance of a nonlinear regression sigmoidal dose response analysis [35]. This step extrapolated protein concentrations for the unknown samples. The antilog (10^x^) of the obtained values was then calculated followed by a multiplication by the dilution factor in order to obtain the accurate protein levels. ROC curves were generated on the same software by listing the measured protein levels in any two-subject groups under comparison.

### AUC Generation for Selecting Multiplexing Candidates

For statistical multiplexing of candidate markers; the list of subjects used was curated to ensure that all the cases and controls included had measurements of a full panel of the biomarker candidates plus ALT. ROC curve comparisons and statistics were conducted on this population using the SAS statistical program (*SAS 9.2, SAS Institute Inc., North Carolina, USA*). For each putative biomarker, ROC curves were plotted and Area Under Curve (AUC) values calculated along with their associated 95% Confidence Intervals (CI) and Standard Error of the Mean (SEM) using the PROC LOGISTIC function and %PLOTROC macro sourced from SAS Institute website (http://support.sas.com/kb/25/018.html). Comparisons of AUC values were conducted using the %ROC macro downloaded from http://support.sas.com/kb/25/017.html.

### Biomarker Combinations at Specified Cut-offs

For each of the protein combinations that demonstrated greater discriminatory power than ALT, optimal cut-offs were calculated based on the maximum likelihood ratio associated with the highest achievable balance in sensitivity and specificity. These indices were calculated for ALT, α1AT, Apo A1, CC3 and HPX on a subset of the GLCS populations using Graph Pad Prism. To further elucidate these results, the cut-offs were applied to the three subject categories, to see what levels of sensitivity and specificity were attainable. Binary combinations based on the “AND” and “OR” methodologies were analysed and resultant sensitivity and specificity indices tabulated. In these binary combinations “AND” and “OR” were defined as follows: “AND” meant that only a positive result on both tests was considered positive and “OR” that only negative results on both tests was considered negative.

## Results

### Main Causative Factors for Chronic Liver Disease Represented in GLCS Population in a Condition-specific Pattern

The GLCS consists of well-defined patient groups representing the main causative factors of Chronic Liver Disease (CLD); HBV, HCV and or AFB1 exposure. In the control group the HBsAg positive rate was measured as 8.4% with none of these individuals showing ‘e’ Antigen positivity, a marker for active viral replication. 5.8% of the 120 controls were HCV antibody positive. In the LC group, 14.4% of patients were HCV antibody positive, 54.5% HBsAg positive and from these 31.7% showed the presence of the HBV ‘e’ antigen. In the HCC sub-group, the HCV positive rate was found to be 31.7%, HBsAg carriage stood at 55.6% and the amount of ‘e’ antigen positive individuals amongst them at 22.6%. The presence of Single Nucleotide Polymorphisms in the p53 gene, used as a marker for aflatoxin exposure [36] were also investigated in the GLCS subjects using Restriction Fragment Length Polymorphism Polymerase Chain Reaction and this showed the presence of at least one mutation in 4.4% of controls, 12.4% of LC’s and 36.2% of subjects diagnosed with HCC. Almost all the point mutations detected were at the codon 249 (table 1) [37].

### Label-free Quantitation Identifies Differentially Expressed Proteins in Plasma between LC, HCC and Control Subjects from GLCS

The levels of protein present in each of the nine pools were found to be of the same order of magnitude (data not shown) thus providing a similar baseline for preparative isoelectric focussing. SDS-PAGE analysis demonstrated fraction-by-fraction profiles, which appeared to be similar between the different disease and healthy control groups with no presence of predominating protein bands. Seven of the total twenty-four isoelectrically focused fractions containing the majority of protein material were subjected to in-solution trypsin digestion and analysed by Q-TOF MS^E^. This yielded 1307 unique protein identifications for the combined 7 processed fractions, with the highest number of protein identifications made at the lanes corresponding approximately to pH 5.5–6.5; (fractions 13, 14 and 15– figure 1A). These results include proteins with condition-specific changes in expression ratios as high as 21 fold (fraction 13) and 22 fold (fraction 15) as well as other proteins exclusively found in specific disease states.

### Stringent Filtering Results in the Identification of 26 Differentially Expressed Proteins from the GLCS Population

A set of 21 extracellular and plasma proteins were identified and shortlisted following streamlined filtering as described in the methods section as differentially expressed in HCC subjects versus individuals with no evidence of liver related ailments. In workflows comparing controls and subjects with active liver cirrhosis, 22 proteins were shortlisted as possible biomarker candidates. The overlap between the two groups means that a total of 26 proteins (table 3) were with the GLCS population found to have condition-specific signatures over the spectrum of non-liver related ailments to the development of liver cancer. Of these 26, alpha-2-macroglobulin, complement factor I, carboxypeptidase N (polypeptide 2) and caspase 8 showed expression patterns suggesting their diagnostic potential for HCC only, whilst the MS^E^ data for complement component 4A, haptoglobin, complement factor B, α1AT and serum amyloid p suggested the changes in these proteins as unique to subjects with LC. As the diagnosis of early stage HCC in a cirrhotic liver is of significant clinical importance, all validated proteins, regardless of which progressive condition they seemed significant or unique for, were measured in all three sub-groups.

### Alpha-1-Antitrypsin, Complement Component 3, Apolipoprotein A1 and Hemopexin show same Expression Trends in MS and ELISA based Analysis

**Figure 2:**
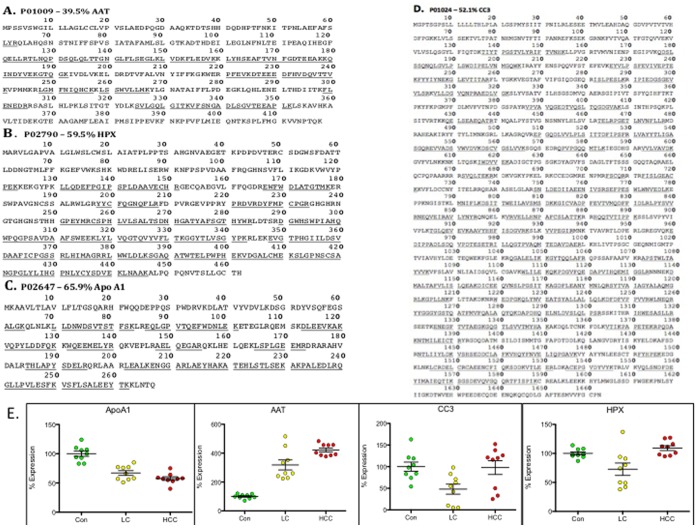
Identification of differentially expressed proteins in plasma from control, cirrhosis and HCC subjects by label-free quantitative MS. (**A–D**) Sequence coverage levels for differentially expressed proteins identified and selected for independent validation. Underlined sequences represent tryptic peptide fragments identified by MS^E^. (**E**) Relative expression plots of robustly identified peptides uniquely attributed to either ApoA1, α1AT, CC3 or HPX and comparing their relative mass peak intensities across the three different disease groups.

**Figure 3:**
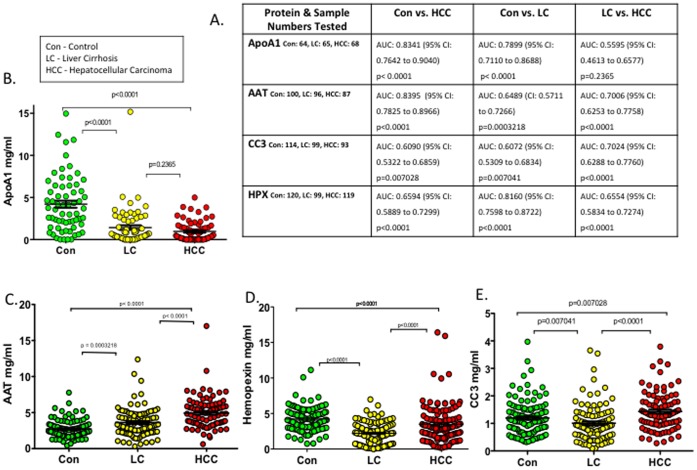
Protein marker validation by ELISA in GLCS. (**A**) Data of sample numbers tested per protein reported with AUC’s, confidence intervals and associated p values. (**B–E**) Dot plots highlighting trends in protein level expression across controls and subjects with LC or HCC for α1AT, Apo A1, CC3 and HPX.

Individual GLCS subject validation by ELISA allowed for direct measurements of target proteins in plasma in order to verify the expression changes suggested by MS. To achieve this, we selected four shortlisted proteins based on representation of various protein families interpreted as suggestive of their involvement in different pathways, implications in literature and availability of quality commercial antibody based methods for direct measurements and took them forth for more comprehensive validation. Plots showing the relative expression trends for three representative peptides in triplicate allocated to α1AT, CC3, Apo A1 and HPX identified with high scores in all three disease categories were selected and compared (figures 2E & 3 B–E). Without exception, the trends in expression seen at the peptide level, between the three disease groups were identical to those seen in direct measurement of these four proteins in individual subjects by ELISA.

### ELISA Validation of Four Markers Identified by Label-free Proteomics Suggests Diagnostic Potential

The four proteins selected for validation were measured in individual GLCS subject plasma across the three disease groupings using ELISA based assays (figure 3). Measurement of α1AT showed a net increase in the proteins overall level with the most significant change observed between mean amounts of plasma α1AT in the control versus HCC groups; an AUC of 0.8395, calculated by ROC analysis suggests a high discriminatory potential. HPX levels in subject plasma showed an overall trend of lowered expression in those with active cirrhosis compared to controls, with a “bounce back” effect seen as the protein levels are somewhat restored to control levels in the HCC subjects. The highest reported AUC was for the change in HPX levels of Con vs. LC; 0.8160. The trend for CC3 expression was similar to that of HPX with its levels dropping from control to LC and rising again in the presence of a liver tumour. However with this protein, the biggest observable difference was seen between cirrhotic and HCC patients with an AUC of 0.7024. The last protein from our shortlist which was validated by ELISA was the high density lipoprotein component, Apo A1. The trend with this candidate was a significantly lowered expression as patients develop LC and HCC. The biggest change in expression was observable between the control and HCC groups with a reported AUC of 0.8341. The strong suppression in ApoA1 levels seen between controls and LC subjects also gave a significant AUC reading of 0.7899 (figure 3).

### Chronic HBV Infection is Strongly Associated with Chronic Liver Disease Development in JUTH Subjects

To an even greater degree than that observed in the GLCS, chronic HBV infection was seen to be strongly associated with the development of LC and HCC in the subjects from Nigeria. Other risk factors for HCC and LC development noted from both subject populations (tables 1 & 2) are being of male sex, increased age, elevated ALT and AFP levels. A low-lying trend of HCV infection is also observable in the NHCC group although it is slightly above the estimated prevalence for West Africa [38]. No assessment of aflatoxin exposure was conducted in this subject population.

### Previously Reported Protein Expression Trends Validated in a Pilot Population of Independent West African Subjects

**Figure 4:**
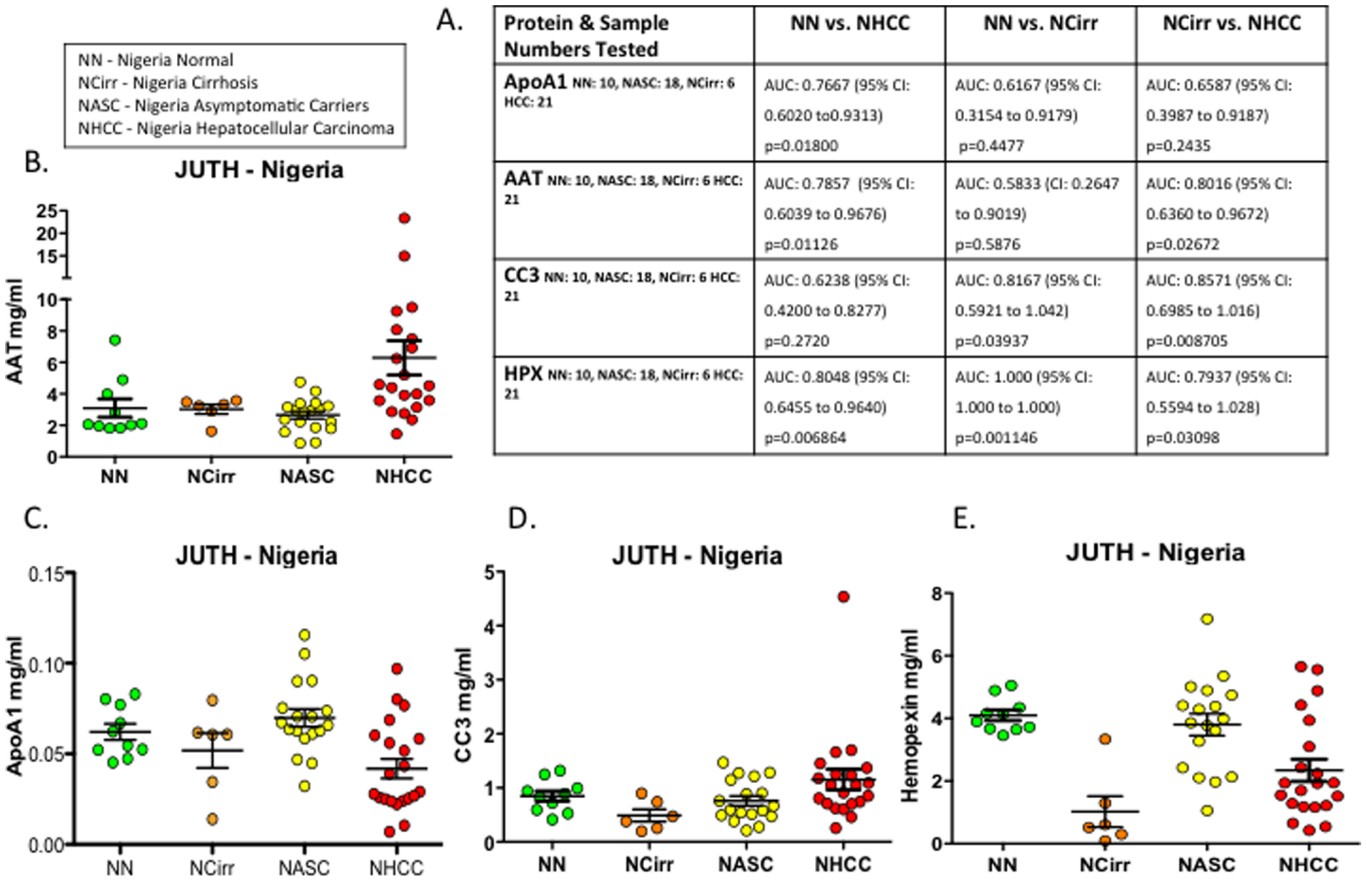
Protein marker validation by ELISA in JUTH. (**A**) Data of sample numbers tested per protein reported with AUC’s, confidence intervals and associated p values. (**B–E**) Dot plots highlighting trends in protein level expression across controls and subjects with Asymptomatic liver disease, LC or HCC for α1AT, Apo A1, CC3 and HPX.

ELISA assays which measured the expression levels of α1AT, Apo A1, CC3 and HPX in the JUTH subjects, showed trends consistent with those seen in the GLCS (figures 3 & 4). In the latter population, α1AT expression was seen to discriminate best between controls and HCC subjects based on AUC calculations. A similar trend was seen in the Nigerian subjects which showed α1AT to give an AUC of 0.7857 (figure 4A.) when comparing overall expression between control and HCC subjects. This was marginally lower than that for NCirr vs. NHCC discrimination with this slight discrepancy in trend likely due to the small number of cirrhotic patients compared to HCCs within the JUTH subject group. The marked decline in ApoA1 plasma secretion previously observed was once again seen, resulting in a peak AUC of 0.7667 between the NN and NHCC groups. Overall, the same trend witnessed in the Gambian subjects was mirrored in those from the JUTH with a marked down-regulation of Apo A1 as liver disease progressed towards hepatocarcinogenesis. The consistency between the two populations was also maintained for CC3 and HPX expression with the highest discriminatory potential for the former seen in the development of HCC from a background of LC. In the Nigerian subjects, this was seen with a peak AUC of 0.8571. With HPX, consistent with previous results, levels of expression were seen to drop in patients with LC and “bounce back” in those with HCC. The calculated AUC for the development of LC in the Nigerian subjects suggests excellent discriminatory potential for this protein with a statistically significant (p.001146) AUC value of 1.000.

### Analysis of Test Combinations Suggest HPX, CC3 and Apo A1 as Superior to ALT in Diagnosing LC

**Figure 5:**
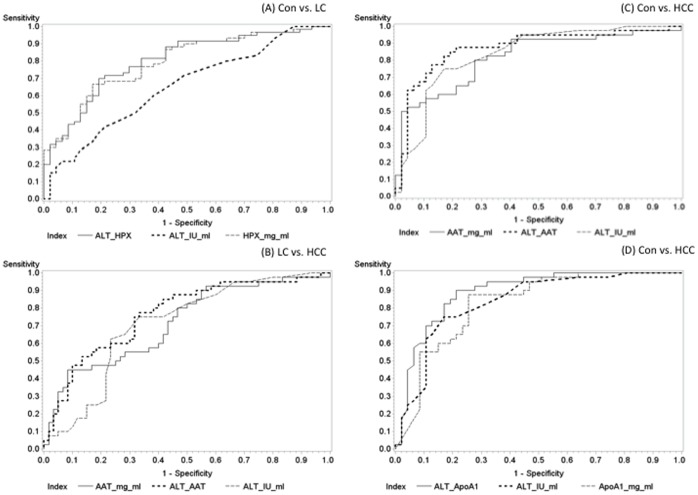
ROC Curves for Multiplexed Proteins in GLCS. (**A–D**) ROC curves demonstrating shifts in discriminatory ability for labeled comparisons between putative markers and ALT.

Comparisons of various combinations of the four validated candidate biomarkers and ALT highlight that HPX, CC3 and Apo A1 are individually all superior to ALT in discriminating control and LC cases. HPX in this subject population has been shown to have an AUC of approximately 0.80 (figure 3A), a value that is significantly higher than that for ALT when used singly to distinguish the same two groups; 0.6507 (figure 5). A negligible boost in diagnostic accuracy from the single use of HPX is seen when these two markers are combined (table 4) although the difference between using HPX and HPX & ALT combined does not reach significance. However, combining ALT and a complex of HPX & ALT offers similar discriminatory power (AUC 0.7949) and carries a significant improvement compared to using ALT alone (p = 0.0095).

**Table 4:** Statistical summary of combinations of putative marker candidates with routine liver function tests.

Con (n = 47) vs. HCC (n = 40)	AUC	SEM	95% CI	Comparison(p)
**ALT (IU/ml)**	0.8372	0.0437	0.7516–0.9228	n/a
**ALT_α1AT**	0.8723	0.0412	0.7915–0.9513	α1AT vs. ALT_ α1AT p = 0.0765ALT vs. ALT_ α1AT p = 0.4288
**ALT_ApoA1**	0.8899	0.0357	0.8199–0.9599	ApoA1 vs. ALT_ ApoA1 p = 0.0183ALT vs. ALT_ApoA1 p = 0.1379
**Con (n = 47) vs. LC (n = 60)**				
**ALT (IU/ml)**	0.6507	0.0532	0.5464–0.7550	n/a
**HPX_ALT**	0.8014	0.0428	0.7176–0.8852	HPX vs. HPX_ALT p = 0.5871ALT vs. HPX_ALT p = 0.0095
**LC (n = 60) vs. HCC (n = 40)**				
**ALT (IU/ml)**	0.7079	0.0524	0.6053–0.8105	n/a
**α1AT_ALT**	0.7663	0.0488	0.6707–0.8618	α1AT vs. ALT_ α1AT p = 0.0899ALT vs. ALT_ α1AT p = 0.2066

The other two proteins suggested to perform better than ALT in detecting LC are CC3 and Apo A1, with respective AUC’s of 0.7039 and 0.7638 (data not shown). In both cases, some discriminatory benefit is indicated by complexing the proteins with ALT alone although the degree of changes seen are not significant. When both proteins are complexed with ALT, the AUC’s obtained improve significantly from that seen with single ALT measurements.

### Analysis of Test Combinations Suggests ALT Combined with α1AT, Apo A1 or HPX Boosts Discriminatory Potential in HCC Patients

ALT in the GLCS population shows high performance (AUC 0.8372) in discriminating the control and HCC groups. Despite this, some benefit is still observed when it is complexed with α1AT or Apo A1 with upwards shifts in AUC to 0.8723 and 0.8899 respectively (figure 5 C & D). The latter combination of ALT & Apo A1 results in noteworthy statistics suggesting that the increase in diagnostic ability conferred by using the two markers in a panel is significant; p = 0.0183 (table 4).

As has been the trend in all the analyses of this study, the two groups most difficult to differentiate are those classed as LC and HCC. From the multiple comparisons performed, two of the markers demonstrated an improvement from the discriminatory values seen with ALT alone. HPX on its own did not perform better than ALT in distinguishing these two groups but when complexed with ALT demonstrates an AUC of 0.7650. A marginal increase is also observed for α1AT over single ALT use but its highest values are attained when used in tandem as seen in table 4.

### Application of Specified Cut-offs to Binary Protein Combinations Significantly Boosts Diagnostic Ability

The application of defined cut-offs to protein combinations with superior AUC values to single ALT use primarily highlighted that the “OR” criteria for test design may be superior in offering balanced sensitivity and specificity values for diagnosis. These results also suggest that higher individual sensitivity or specificity values are attainable with the “AND” criterion.

ALT combined with CC3 in discriminating CON and LC subjects; HPX for LC and HCC and ApoA1 in CON and HCC discrimination showed upward shifts in AUC with significant p values, though the significance level for the first comparison was borderline (p<0.0687). When specified cut-offs were used, accurate subject assignments between the CON and HCC groups with the following criteria – “ALT of >10.50 IU/ml OR ApoA1>0.7673 mg/ml” had respective sensitivity and specificity values of 82.5% and 74.5%. HPX & ALT combined with the same OR criterion at cut-offs of HPX>2.701 mg/ml and ALT>13.50 IU/ml in the LC and HCC categories had high sensitivity, but poorer specificity; 97.50% and 51.67% respectively (Table S1A & C).

In the use of ALT and CC3 together for discerning the CON and LC groups, the “OR” command again showed a greater balance in achievable sensitivity and specificity; 85.0% & 57.5%, respectively than using the cut-offs of ALT>9.5 IU/ml and CC3<0.9579 mg/ml with the “AND” command. The latter produced results which skewed the balance towards poor sensitivity (18.33%) but high specificity (93.62%) (Table S1b).

## Discussion

As with many solid tumours for which high performance non-invasive diagnostic tools are lacking, the identification of reliable predictive markers for HCC is of prime importance. The greatest burden of this disease and its major causative factors exist in the developing world where many of the sophisticated imaging tools used to compensate for the insufficient performance of AFP are not widely available. The liver, as the main site of plasma protein synthesis and metabolic activities such as detoxification and storage is a central organ in the body. Measurement of plasma proteins, even the highly abundant acute phase proteins, most of which are processed by or derived from it, can as such provide deep and relevant insight into how specific changes in key proteins may be relevant in the development of HCC.

To address the issue of heterogeneity and sample selection bias which has the potential to impact all human studies, a large population of 339 subjects was selected and pooled as to average out individual differences and highlight more general overarching trends. Suspected aetiology of liver disease and age were considered as the basis for stratifying and pooling samples but the latter was chosen as it offered a more even distribution of subjects, per sub-group and would allow for a more distinct separation strategy, as many of the subjects were positive for more than one of the key aetiological factors (HBV, HCV or p53 mutation). There is also a growing body of work which suggests that there are age related changes which occur in the liver resulting in its reduced volume, decline in metabolism of certain drugs, changes in protein expression and lowered hepatobiliary functions. Other more subtle changes have also been linked to the ageing liver, particularly at the DNA level and are considered to result in lowered rates of DNA repair, which may have direct implications in virus, or toxin induced mutations that lead to hepatocarcinogenesis [39], [40]. The pooling of samples by age within the distinct disease categories encouraged the extraction of proteins whose secretory and regulatory signatures are not age dependent. Candidates robust enough to remain highlighted through this process stand at some advantage, as the observation of uniform trends across the three age ranges suggest they will likely be usable at all levels of healthy or disease liver function.

Direct measurement of Apo A1 by ELISA in individual GLCS subjects (figure 3B) confirms the changes in protein abundance predicted by MS and shows the protein to be severely down-regulated with impaired liver function as determined by deteriorating liver enzyme readings (table 1). This finding suggests that changes in its secretory pathway are altered during disease progression. Other studies [41], [42] corroborate these findings with one group proposing the lowered expression of Apo A1 in subjects with confirmed HCC as a prognostic marker for portal vein metastases [43]. α1AT by comparison shows a reverse trend with increasing circulatory plasma levels as liver function wanes. The serpin protease and its precursor fragments have been implicated in numerous LC and HCC related studies as plausible biomarker candidates. One such study linked α1AT expression to treatment efficiency following Transarterial Chemoembolization (TACE) [44]. Kang and colleagues in a LC-MS based investigative screening exercise looking at sera from 9 histologically confirmed HCC’s compared with LC subjects reported a 10-fold change in α1AT expression between the two groups [45]. α1AT as a major plasma glycoprotein with three reported glycosylation sites [46] has had its core fucosylated form [47] also specifically shown to have altered modification patterns that are highly sensitive and specific to HCC and or LC [48].

Direct ELISA measurements of CC3 and HPX in individual subjects show a dip in expression with the development of LC and a restoration to healthy levels of protein expression within the HCC group. This pattern may be indicative of a loss of liver functionality with LC due to a larger volume of the liver being affected, whereas hepatocarcinogenesis on a liver without a background of extensive cirrhosis could have greater overall functionality and hence still be able to produce key proteins at customary levels.

The identification of these two proteins as strongly associated with chronic liver disease (CLD) is consistent with other studies which demonstrate that CC3 levels or those of its processed units alter significantly between individuals with HCC in comparison to healthy controls or CLD sufferers [42], [49], [50]. MALDI-TOF MS profiling of serum from 78 HCC cases matched with 72 cancer free controls from Egypt identified six candidate markers showing strong association with HCC, regardless of hepatitis virus status. One of the panel of six was identified by sequencing as a fragment of CC3 [51]. A similar study conducted in Japan on HCV associated HCC versus HCV related CLD without hepatotumorigenesis identified the protein fragment demonstrating the most significant differential expression between the two groups as belonging to CC3 [52]. Similar reports have been made for our fourth validation marker; hemopexin. A study, which measured levels of fucosylated HPX found significantly, elevated quantities of its expression in a cohort of 229 serum samples from patients with chronic hepatitis, LC, and HCC [53]; a finding that was previously reported in an earlier publication [54]. A recent study by Japanese investigators also assessing the clinical utility of serum fucosylated hemopexin in HCC, LC and chronic hepatitis subjects concluded that the glycoprotein could be used as a biomarker potentially indicative of a hypercarcinogenic liver [55].

The trends reported from the quantitative measurements of all four shortlisted proteins conducted by ELISA in the pilot validatory JUTH subjects show expression trends largely identical to those seen in the Gambian group. These experiments independently confirm that the secretion of Apo A1 into plasma is increasingly inhibited during LC and HCC development; α1AT levels are marginally increased with LC followed by a marked up-regulation with progression to HCC. CC3 & HPX show alternative trends to the afore mentioned markers suggesting them to possess signatures more exploitable for the diagnosis of LC or HCC from a cirrhotic background than that from a healthy non-cirrhotic liver. It is worth noting though that the distinction of the first two groups would be of greatest clinical relevance as up to 80–90% of HCCs have been reported to develop from a background of LC [56] making it a primary risk factor for HCC onset. The differences in discriminatory power shown by these proteins suggest their involvement in alternate pathways; an implication that could prove particularly useful in their utilization as part of a panel of multiplexed markers with calculated cut-offs for diagnoses based on achievable sensitivity and specificity. In comparing our findings with reports from other studies; specific alterations in target protein trends may be identified; these will likely be attributable to the different subject populations under study, host genetics, dominant aetiological factor associated with LC and HCC in any given populace as well as the role played by confounding factors such as aflatoxins. Significant differences in the trends of expression of biomarker candidates may also be due to the stringency of case definitions and diagnostic parameters as well as varying study designs.

In any attempt to identify a viable, high performance biomarker within a subject population; shortlisted candidates will at some point have to be compared against contemporary tests in current clinical use. A major challenge faced in doing this is when these benchmark assays have been used intrinsically to classify the various subject groups under study. AFP formed part of the diagnostic profile of both the GLCS and JUTH subjects – thus when used in these populations to approximate its diagnostic ability, the observed AUC was grossly exaggerated. Consequently, when it came to the utilization of statistical methods to combine α1AT, Apo A1, CC3 and HPX with more established markers – an independent indicator of liver health, ALT, had to be employed. It must be noted however that the measurement of this enzyme is not irrelevant to the subject of chronic liver disease diagnosis especially amongst long-term HBV carriers in whom studies have shown the level of ALT to be associated with elevated risk of morbidity and poor development of liver health [57], [58].

Patients positive for the HBeAg are considered to be at an advanced risk for HCC development due to its indication of active viral replication; within this group, rising ALT is also associated with increased likelihood of seroconversion [59]. A large study including 3160 participants in Taiwan tried to determine the effect of ALT in defining HCC risk. After a follow up period of 38,330 person years in which 81 participants developed HCC, it was concluded that trends in long-term levels of ALT expression was an independent predictor of HCC development and its regular monitoring in chronic HBV carriers is of clinical important [60]. In light of these reports, the choice of ALT as a comparative marker in this investigation may not be ideal but is still useful in offering insight as to how these four proteins perform in comparison to current routine clinical indicators of liver disease. Not much has been reported on the independent diagnostic ability of AFP, but reports approximate it at 0.70, with at least one publication showing AUC values as low as <0.60 when used at a cut off of 100 ng/ml [61].

The results detailed above highlight the protein combinations which perform better than the sole use of ALT and AFP approximation. The shifts seen with the application of calculated cut-offs depict the fine balance that has to be struck when combining multiple proteins into a single diagnostic test. No single approach to this has been universally proposed; rather the logic for how multiple tests are combined continues to be uniquely tailored to the complimentary values of the markers under use. To begin to move these combination methods toward successful application in real life assays, some synergy will need to be established between medical researchers who identify and propose candidates and kit developers with the expertise to design viable pilot assays useable in the extensive validation of novel test panels.

The attempt made in these investigations to advance differentially expressed proteins from discovery experiments and validate their expression profiles in a regional subject group with expected similar aetiology of liver disease is an important step in establishing the differential expression and robustness of these markers. The major findings detailed carry the potential for further investigations helping to characterize how these proposed HCC and LC biomarkers may perform in non-African chronic liver disease subjects as well as a more thorough description of possible mechanisms at play in bringing about the described expression changes. Such investigations are warranted but will require extensive resources and expertise as well as collaborations between international laboratories in order to promote candidates to a point where they can be strongly proposed for development into routine laboratory assays.

## Supporting Information

Figure S1 **Scheme summarizing how GLCS subjects were categorized into the three clinical groups following informed consent.**
(TIFF)

Figure S2 **Scheme of workflow detailing major steps undertaken form discovery and quantitative proteomics to independent validation.**
(TIFF)

Table S1 **(A–C) Statistical summary from the combination of proteins using select cut-offs as a measure of diagnostic potential demonstrated by achievable sensitivity and specificity, for the Con, LC HCC groups from the GLCS.**
(TIFF)
