# Correction: Protein Profiling in Hepatocellular Carcinoma by Label-Free Quantitative Proteomics in Two West African Populations

**DOI:** 10.1371/annotation/5330d029-ab4d-447d-837f-99b965956234

**Published:** 2013-09-11

**Authors:** Haddy K. S. Fye, Cynthia Wright-Drakesmith, Holger B. Kramer, Suzi Camey, Andre Nogueira da Costa, Adam Jeng, Alasana Bah, Gregory D. Kirk, Mohamed I. F. Sharif, Nimzing G. Ladep, Edith Okeke, Pierre Hainaut, Simon D. Taylor-Robinson, Benedikt M. Kessler, Maimuna E. Mendy

In the citation, the name of the fifth author was presented incorrectly.

The correct citation should read: Fye HKS, Wright-Drakesmith C, Kramer HB, Camey S, Nogueira da Costa, A, et al. (2013) Protein Profiling in Hepatocellular Carcinoma by Label-Free Quantitative Proteomics in Two West African Populations. PLoS ONE 8(7): e68381. doi:10.1371/journal.pone.0068381 

