# Supplementary figures and images for: Protein Profiling in Hepatocellular Carcinoma by Label-Free Quantitative Proteomics in Two West African Populations
Source: PLoS One. 2013 Jul 30;8(7):e68381. doi: 10.1371/journal.pone.0068381 (PMC3728326; doi:10.1371/journal.pone.0068381)

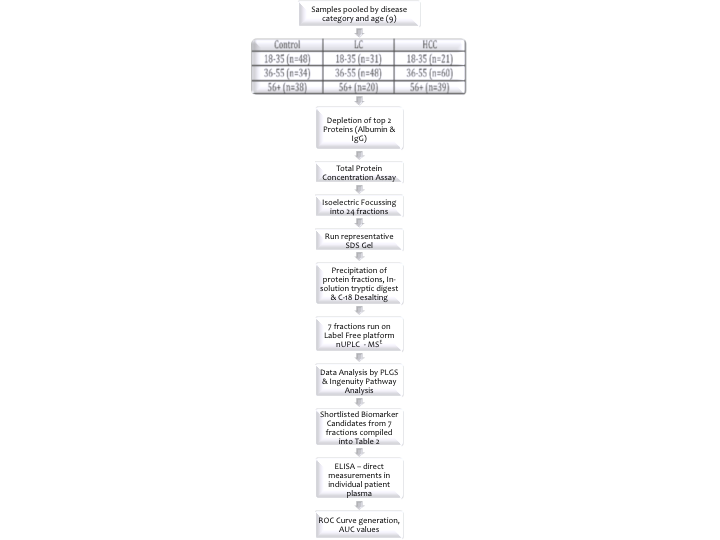

Supplement: Figure S2 — Scheme of workflow detailing major steps undertaken form discovery and quantitative proteomics to independent validation. (TIFF) [file pone.0068381.s002.tiff]
